# Evidence-Informed Policy-Making: Will It Ever Be Enough? A Response to the Recent Commentary

**DOI:** 10.34172/ijhpm.2022.7722

**Published:** 2022-11-01

**Authors:** Sultana Al Sabahi

**Affiliations:** Directorate General of Planning and Studies, Ministry of Health, Muscat, Oman.

 In their commentary, Ellen and Ben-Sheleg highlighted the need for doing more in the field of evidence-informed policy-making (EIPM). They emphasized the importance of monitoring and evaluating EIPM activities and the need to do more to take the field to the next level. The authors were encouraging a more innovative approach to evaluate the impact of EIPM, to avoid reinventing the wheel and bring more from relevant fields like behavioural economics, psychology, and marketing in getting the individuals and organizations to change their behaviour, and upscaling the opportunity that has been offered by the coronavirus disease 2019 (COVID-19) pandemic. The commentary also suggested developing a gradient to prioritize which issue of healthcare concerns could be solved through evidence-informed policy. While this idea might be fascinating to increase policy-makers’ buy-in, it also encourages us to ask whether we can ever say that we have done enough in the EIPM field. We need to ask if EIPM can ever be 100% evidence-based as Ellen and Ben-Sheleg have tried to. How to be strategic in selecting which healthcare issue to solve? What is the importance of the context when creating short-term wins? And what are the special characteristics of the health sector that need to be considered when borrowing from other field into the EIPM?

## Evidence-Informed Policy-Making: Can It Ever Be Enough?

 An essential role of EIPM is to strengthen the importance of evidence in decision-making in healthcare, which is increasingly complex given the exponential growth of research and information. EIPM is only a few decades old, yet it has accomplished a lot so far. Although the main approach that EIPM advocates have used was policy brief and policy dialogue, it evolved dramatically and showed great agility and flexibility to accommodate the unpredictable world. For instance, during the COVID-19 pandemic, McMaster University constructed the COVID-19 Evidence Network to support Decision-making (COVID-END) at the start of the pandemic to cope with COVID-19 by using the best available evidence.^1^ In 2021, COVID-END convened the Global Commission on Evidence to Address Societal Challenges to change the global panorama on evidence generation. This led to the publication of a report titled “A wake-up call and path forward for decision-makers, evidence intermediaries, and impact-oriented evidence producers.”^2^ In addition, Cochrane published “Cochrane Convenes: Preparing for and responding to global health emergencies. Learnings from the COVID-19 evidence response and recommendations for the future.”^3^ Cochrane brought together leaders in health research and health evidence to explore and recommend the changes needed in evidence synthesis to prepare for and respond to future global health emergencies. These are a few examples of rapid improvement in the EIPM initiatives. It clearly indicates that, given the complex nature of healthcare and policy-making, it is hard to predict which direction EIPM might take. However, its agility and ability to adapt to rapid change to provide the needed support for policy-makers indicate that we might not reach a point where we can say that this field is doing enough because each achievement will highlight the need to do just more in a different dimension.

## The Battle That Had Never Been Completed: How Should It Be Ended?

 Ellen and Ben-Sheleg highlighted that EIPM scientists are failing to monitor and evaluate EIPM initiatives’ impact.^4^ Although all scientists would love to see the impact of their initiatives on the policies and decisions, claiming simple achievement of this goal will be questionable given the complexity of policy-making. It is important to remember that the effects of policies are often indirect, diffuse, and take time to appear.^5^ Policies cannot be presented as discrete interventions to tackle specific problems, whose effects can then be reliably measured and evaluated. It has been reported that policies are always influenced by many other factors other than evidence.^6^ There is a high chance that a policy will produce an effect that is not measurable and not attributable. In addition, we should keep in mind that a policy impact might be cumulative of many different initiatives. Therefore, unless the impact and influences of other interlinked initiatives are well considered, a policy’s real impact cannot be properly understood. Given this complex nature of policy-making, the weak achievement of EIPM in monitoring and evaluation should not be superficially attributed to this field. Instead, it should go deeper into the political nature of policy-making. Hence, we might realize that there is not so much a lack of recommendation for monitoring and evaluation, just a lack of realistic conceptualization.

## Short Wins Do Matter to Support EIPM in a Particular Context

 In the original paper,^7^ we reported that different countries took different approaches to advocate for better utilization of evidence in the policy-making process. These approaches might be setting the priority, proof of concept, building capacity, or writing a policy brief. While looking from the outside and comparing to countries that are very advance in the EIPM field, such efforts might be tangible and not enough to reach the intended change. Yet the impact of such initiatives is reported to be dramatic in creating a case of success, clarifying the concept, and getting the buy-in from policy-makers and stakeholders.^7^ Therefore, the short-term wins should not be underestimated, and evaluating its role in making the change should always be tightly connected to the context where the initiative is taking place. Hence, we should ask if we need a major shock to the system to initiate change. Or can there be incremental change?

## Looking Outside the EIPM World

 Ellen and Ben-Sheleg touched on the importance of utilizing the advancement in the other filed, such as behavioral economics and theory of change. While the essence of knowledge translation is connected to different social science theories (eg, Diffusion of Innovation Theory, Agency theory, Situated-change theory, and Institutional theory),^8^ more can be utilized from the implementation science to advance the EIPM field. It is anticipated that knowledge translation and implementation science can be leveraged to advance the EIPM by improving the fit between problems and approaches to implantation through a theory-driven, staged, and systematic approach that integrates knowledge translation principles and processes and involves key stakeholders’ interest.^9^

## Ethical issues

 Not applicable.

## Competing interests

 Author declares that she has no competing interests.

## Author’s contribution

 SAS is the single author of the paper.
